# Associations Between Infant Negative Affect and Parent Anxiety Symptoms are Bidirectional: Evidence from Mothers and Fathers

**DOI:** 10.3389/fpsyg.2015.01875

**Published:** 2015-12-02

**Authors:** Rebecca J. Brooker, Jenae M. Neiderhiser, Leslie D. Leve, Daniel S. Shaw, Laura V. Scaramella, David Reiss

**Affiliations:** ^1^Department of Psychology, Montana State UniversityBozeman, MT, USA; ^2^Department of Psychology, The Pennsylvania State UniversityUniversity Park, PA, USA; ^3^Department of Counseling Psychology and Human Services, University of OregonEugene, OR, USA; ^4^Department of Psychology, University of PittsburghPittsburgh, PA, USA; ^5^Department of Psychology, University of New OrleansNew Orleans, LA, USA; ^6^Yale Child Study Center, Yale UniversityNew Haven, CT, USA

**Keywords:** infancy, mother, father, negative affect, anxiety risk

## Abstract

Little is known about child-based effects on parents’ anxiety symptoms early in life despite the possibility that child characteristics may contribute to the quality of the early environment and children’s own long-term risk for psychological disorder. We examined bidirectional effects between parent anxiety symptoms and infant negative affect using a prospective adoption design. Infant negative affect and adoptive parent anxiety symptoms were assessed at child ages 9, 18, and 27 months. Birth parent negative affect was assessed at child age 18 months. More anxiety symptoms in adoptive parents at child age 9 months predicted more negative affect in infants 9 months later. More infant negative affect at child age 9 months predicted more anxiety symptoms in adoptive parents 18 months later. Patterns of results did not differ for adoptive mothers and adoptive fathers. Birth parent negative affect was unrelated to infant or adoptive parent measures. Consistent with expectations, associations between infant negative affect and rearing parents’ anxiety symptoms appear to be bidirectional. In addition to traditional parent-to-child effects, our results suggest that infants’ characteristics may contribute to parent qualities that are known to impact childhood outcomes.

## Introduction

High levels of negative affect and withdrawal during infancy are linked to increased odds that children will receive an anxiety diagnosis prior to adolescence (Clauss and Blackford, 2012). High levels of anxiety symptoms in parents appear to compound early risk for disorder such that the offspring of more anxious parents display more negative affect (Rosenbaum et al., 1988) and are at greater risk for developing anxiety problems relative to offspring of non-anxious parents (Beidel and Turner, 1997). However, a strict “parent-to-infant,” view of early risk oversimplifies the complexities of parent–child interactions (Bell, 1968). Positive associations between parent anxiety symptoms and infant negativity may also reflect an effect of infant characteristics on parents’ anxiety levels. Yet, little is understood about how infants’ negative affect might impact levels of parental anxiety. Empirically testing this putatively bidirectional association holds implications for long-term outcomes in both parents and infants. In the current study, we use a prospective, parent-offspring adoption design that allows for the differentiation between genetic and environmental contributions of parent anxiety to infant negative affect. At the same time, we test infant negative affect as a precursor to levels of mothers’ and fathers’ anxiety symptoms across infancy.

Developmental research has demonstrated that high levels of reactivity, withdrawal, and negative affect early in life are linked to an increased risk for a variety of behavior problems, including anxiety disorders, from infancy through adolescence (Eisenberg et al., 1996; Hane et al., 2008). This link is particularly robust for early fearful negativity, which has been associated with risk for anxiety problems during infancy (Kagan, 1984), toddlerhood (Buss, 2011), childhood (Hirshfeld et al., 1992), and adolescence (Schwartz et al., 1999). Constructs that have been previously used to assess negative affect as a risk factor for anxiety problems vary in the degree to which they focus on fear or on broader constructs of negativity. Because we recognize some, albeit incomplete, overlap across these constructs (e.g., fearfulness, difficult temperament, behavioral inhibition, negativity, etc.), we refer cumulatively to these dimensions as negative affect in this report. Similarly, we highlight the fear dimension of negative affect given its robust associations with anxiety risk in previous research; however, because different theoretical perspectives exist regarding the degree to which emotions are differentiated early in life (Izard, 1978; Lewis and Michalson, 1983), we do not exclude other dimensions of negative affect.

Greater anxiety symptoms in mothers during pregnancy and across early development are linked to more behavioral and emotional problems in children (Beidel and Turner, 1997; O’Connor et al., 2002). Likewise, children of anxious parents are more likely to show high levels of negative affect (Rosenbaum et al., 1988) and to develop behavior problems than are the children of non-anxious parents (Weissman et al., 1984; Biederman et al., 1991; Natsuaki et al., 2013). However, there remains a dearth of research on the influence of child behaviors on parent anxiety. Such effects have been implied by findings from work with older children showing that efficacious treatments for anxiety problems led to improvements in parenting behaviors (Silverman et al., 2009). Yet, almost nothing is known about how early negative affect might impact anxiety symptoms in mothers. The possibility of this association is important because, if present, infants may evoke symptoms in parents that will continue to impact their own outcomes over time. Under these conditions, current conceptual models that account only for parent-based effects on children’s risk for anxiety are incomplete. Likewise, intervention efforts aimed solely at reducing parent symptoms ignore children’s potential impact on their own development via influences on parent mental health and early caregiving. Therefore, testing the reciprocal influences of infants’ negative affect and parents’ anxiety symptoms was a primary goal of the current study.

Efforts to fully characterize bidirectional effects between parent anxiety symptoms and risk for anxiety problems in early life would further benefit from an understanding of similarities and differences across mother–infant and father–infant associations. Although limited, evidence exists that children’s behaviors are more strongly linked to mental health in mothers than to mental health in fathers (Hastings and Brown, 2002). Such findings may indicate either that the mother–child relationship has features that are unique from father–child interactions or that fathers are less involved with children, particularly during infancy, than are mothers (Bulanda, 2004). Alternatively, fathers may also be involved with their children in ways that are simply different from mothers (Fitzgerald, 1977; Labrell, 1994; Parke, 1996). At least one study has suggested that anxiety symptoms in mothers and fathers cumulatively increase children’s risk for developing an anxiety disorder (Dierker et al., 1999), though other work has reported null associations between paternal anxiety symptoms and child behaviors (McClure et al., 2001). Given mixed findings on the association between father–child behaviors, a secondary aim of this study was to test for similarities and differences in reciprocal influences of infants’ negative affect and anxiety symptoms in mothers and fathers.

Most parents and children share genetic propensities for mental health outcomes, though infant behaviors likely remain critical elements of early risk. For example, child-based effects on parent symptoms and behaviors in other domains are not fully explained by shared genetic liabilities (Field et al., 1987; McAdams et al., 2015) between parents and children. Nonetheless, genetic factors remain important in the consideration of ways that parents’ and infants’ behaviors may be associated. Specifically, this process by which infants shape their own environments via heritable characteristics can be described as gene-environment correlation (*r*GE; Scarr and McCartney, 1983). *Evocative r*GE arises when genetically influenced traits (e.g., infant negative affect) evoke a response from the environment (e.g., parents’ anxious behaviors). *Passive rGE* occurs when the environment is correlated with children’s genes because, as is the case in most studies, parents and children share both their genes and environments (Plomin et al., 1977). Passive *r*GE acts as a confound in these studies because genetic and environmental mechanisms cannot be disentangled. However, the prospective adoption design used in the current study eliminates the passive *r*GE confound (Leve et al., 2013). Specifically, associations between early negative affect in infants and subsequent parent symptoms cannot be attributed to shared genetic liabilities because children do not share genes with adoptive parents. The prospective adoption design also enables evocative *r*GE to be examined directly: when prenatal factors are controlled, associations between birth parent and child characteristics indicate inherited effects because birth parents are not a part of the child’s environment. Through this design, we can begin to depict not only the presence or absence of child-based effects on parent symptoms, but also the basis of such effects in the child’s genetic makeup.

In sum, we tested bidirectional parent–child effects between two established factors of early risk for anxiety problems: children’s negative affect and parent anxiety symptoms. We included measures of birth parent negative affect, which allowed us to control for one domain of heritable effects, and included adoptive parents, adopted children, and birth parents in our analyses and to directly examine evocative *r*GE. We used a longitudinal, cross-lagged approach to assess associations between parent symptoms and child negative affect across infancy and toddlerhood. We focused on the infant-toddler period based on evidence for associations between children’s negative affect and anxiety outcomes during this time (Biederman et al., 2001; Chronis-Tuscano et al., 2009). Additionally, we focused on typically developing families based on evidence for the importance of subthreshold anxiety symptoms for long-term anxiety outcomes (Karsten et al., 2011). Consistent with previous research, we expected that greater anxiety symptoms in adoptive parents would predict greater negative affect in infants over time. However, we also hypothesized that greater infant negative affect would predict increases in anxiety symptoms in both adoptive mothers and fathers.

## Materials and Methods

### Participants

Participants were drawn from the first cohort of the Early Growth and Development Study (EGDS). EGDS is a multisite longitudinal study of adopted children and their birth and adoptive parents in the US (Leve et al., 2013). To participate, infants had to be: adopted domestically, placed in adoptive homes within 3 months of birth (*M* = 7.11 days; *SD* = 13.28), genetically unrelated to their adoptive family, and physically healthy. In the analytic sample for the current report, 92.1% of infants had been placed into their adoptive homes within 30 days of birth and all infants had been placed into adoptive homes within 72 days of birth. All parents had to comprehend English at the eighth-grade level. The current sample included all families with available infant observational data *and* parent (adoptive parents and birth mothers) self-report data (*N* = 349). Available data from birth fathers was not an inclusion criterion, but were included when available (*n* = 86). This study was conducted with approval from the Human Subjects committee of the Institutional Review Boards of all participating institutions. All Participants provided written informed consent prior to participation, in accordance with the Declaration of Helsinki. Adoptive parents provided consent for children to participate.

In the full sample, most birth mothers (71.1%) were Caucasian (African–American = 11.4%, American Indian/Alaska Native = 2.8%, Asian–American = 1.9%, Native Hawaiian/Pacific Islander = 0.3%, >1 race = 5.0%, Unknown = 0.8%; 6.7% Hispanic), as were birth fathers (Caucasian = 74.6%, African–American = 8.7%, American Indian/Alaska Native = 0.8%, >1 race = 4.8%, Unknown = 2.4%; 8.7% Hispanic). Birth parents most frequently reported high school as their highest education level (mothers = 36.8%, fathers = 41.8%) and annual earnings under $15,000 (mothers = 43.7%, fathers = 42.1%).

Most adoptive mothers (91.4%) were Caucasian (African–American = 3.6%, American Indian/Alaska Native = 0.3%, Asian–American = 0.6%, >1 race = 1.1%; 2.5% Hispanic), as were adoptive fathers (Caucasian = 90.2%, African–American = 5.0%, Asian–American = 0.6%, >1 race = 1.1%; 1.7% Hispanic). Nearly half of adoptive mothers (42.7%) reported a 4-year college degree as their highest level of education; 37.6% of adoptive fathers reported earning a graduate degree. Adoptive families largely reported annual earnings over $100,000 (53.0%). There were no demographic differences between the full sample and the analytic sample.

### Procedure

#### Adoptive Parent Anxiety Symptoms

Adoptive parent anxiety symptoms were assessed using the Beck Anxiety Inventory (BAI; Beck and Steer, 1983) at child ages 9, 18, and 27 months. Adoptive mothers and fathers completed 21 items indicating their experience of different anxiety symptoms. Individual items were summed to create an overall score (mothers: αs = 0.75–0.83; fathers: α = 0.73–0.80).

#### Negative Affect in Birth Parents

Birth parents self-reported trait-level negative affect at child age 18 months using the 26-item Negative Affect scale of the Adult Temperament Questionnaire (Evans and Rothbart, 2007). Individual items were averaged into an overall score (mothers: α = 0.81, fathers: α = 0.80). Because children may inherit genetic liabilities from either birth parent, we quantified genetically based risk as the maximum score across birth parents. This calculation method is consistent with previous work (Brooker et al., 2014). When birth father data were unavailable, only the birth mother’s score was used.

Birth parent negative affect was assessed in the current study because of its close approximation to our measure of risk in children. Similarities across constructs increase the likelihood that contributions from birth parent to infant characteristics will be evident. Notably, substituting birth parent anxiety symptoms (BAI) for birth parent negative affect, which produces greater construct equivalence between birth and adoptive parent measures, resulted in an identical pattern of results.

#### Infant Negative Affect

As noted above, given prior differences in assessments of negative affect and high levels of overlap between fear and other negative emotions early in life, we used a broadly defined composite of negative affect during infancy. Given that parent-reports and observational measures are believed to capture unique aspects of children’s characteristics (Zentner and Bates, 2008) we constructed a multi-method composite of child negative affect.

##### Parent reports

At child ages 9, 18, and 27 months, adoptive parents reported infant negative affect using the fussiness/difficulty subscales of the Infant Characteristics Questionnaire (IBQ; Bates et al., 1979). To ease participant burden, less-reliable or developmentally inappropriate items were removed. Individual items were summed to create scale scores (mothers: α = 0.80–0.81; fathers: α = 0.81–0.82). Maternal and paternal ratings were averaged to reduce rater bias (9-months: *r* = 0.69, *p* < 0.01, 18-months: *r* = 0.56, *p* < 0.01, 27-months: *r* = 0.54, *p* < 0.01).

At child ages 9, 18, and 27 months, adoptive parents rated infants’ fearfulness and fear reactivity. At 9 months, parents rated infant distress to novelty and limitations using the Infant Behavior Questionnaire (Rothbart, 1981). Scale scores were created by averaging individual items (novelty: mother α = 0.73, father α = 0.71; limitations: mother and father = 0.85) and parent ratings were averaged to reduce rater bias (novelty: *r* = 0.51, *p* < 0.01; limitations: *r* = 0.60, *p* < 0.01). At 18 and 27 months, adoptive parents rated infant fearfulness using the social fear subscale (*n* = 19) of the Toddler Behavior Assessment Questionnaire (TBAQ; Goldsmith, 1996) which extends the IBQ scales used here. Scale scores were created by averaging individual items (mother: αs = 0.87–0.93; father: αs = 0.86–0.89). Parent ratings were averaged to reduce rater bias (18 months: *r* = 0.62, *p* < 0.01; 27 months: *r* = 0.57, *p* < 0.01). The scales of the IBQ and TBAQ have demonstrated construct equivalence over time (Goldsmith, 1996) and have been used to model longitudinal development (Brooker et al., 2013).

##### Observed negative affect

At home visits when infants were 9, 18, and 27 months old, children participated in a 2-min interaction designed to assess reactivity to social novelty (Goldsmith and Rothbart, 1999a,b) First, a stranger sat quietly and neutrally on the floor near the child (30 s) before building a tower of stacked cups (30 s). The stranger then invited the child to play (30 s) and, finally, fully interacted with the child by smiling and encouraging him/her to participate in building and knocking down the tower (30 s).

At each assessment, infants also participated in interactions designed to measure reactivity to non-social novelty (Goldsmith and Rothbart, 1999b). At the 9-months, the interaction lasted for 90 s; first, the child was presented with a novel toy that made noises and moved around in unpredictable ways (60 s; *Bumble Ball*; Otis and Claude). The child was then allowed to play with the toy.

At subsequent assessments, a remote-controlled toy dinosaur (18-months) or robot (27-months) was placed in front of the child (Goldsmith and Rothbart, 1999a). The toy was moved forward, stopped 15 cm directly in front of the child, and remained still for 10 s. The toy was then moved back to its starting position where it remained stationary for 5 s. This movement sequence was repeated three times.

#### Data Reduction

Videotapes of the stranger interaction were scored using a previously established coding scheme (Kochanska, 1991; Shaw et al., 2002). Infants were rated on 4-point scales for their avoidance, inhibition to the stranger, inhibition to objects, fearlessness (reversed), active exploration (reversed), and proximity to caregiver during the episode. Scores were assigned in 30-s intervals and then collapsed across the interaction.

Infants’ avoidance of the bumble ball was scored by a separate coding team using a previously established coding scheme (Dogan et al., 2005, Unpublished coding manual.) Children were rated on a 9-point scale for their avoidance of the toy during the episode. Infants’ avoidance during the Dinosaur and Robot episodes was scored on 4-point scales for their inhibition to objects, fearlessness (reversed), active exploration (reversed), and proximity to caregiver during the episode. Scores were assigned in 30-s intervals and collapsed across the interaction.

Four independent coders assigned ratings for each episode. Each coder was required to achieve a minimum reliability of Pearson’s *r* = 0.85 with a master coder before coding independently. Videos were double coded (15%) to calculate inter-rater reliabilities and prevent drift. Coding reliabilities for 9-month behaviors ranged from 0.90 to 0.98 (mean ICC = 0.94) and from 0.79 to 0.94 (mean ICC = 0.91) at 18 and 27 months.

As different numbers of observational and parent-report variables would produce unequal factor loadings in a structural equation model (SEM), we created a single manifest composite variable for infant negative affect at each assessment. First, all variables from observational coding were entered into a Principal Components Analysis (PCA). Single factors accounted for a high proportion of variance in the original variables at 9 (47.99%), 18 (70.27%), and 27 months (66.34%). All factors had high loadings (|mean| = 0.79) and reflected low levels of approach and high levels of negativity, avoidance, and inhibition. Second, factor scores of infant negativity were extracted from the PCA and aggregated with *z*-scored scale composites from parent-report measures to create a single score of infant negative affect at each age.

### Covariates

#### Pregnancy Complications

Self-report measures collected 4-months postpartum were used to score pregnancy and delivery complications. Birth mothers reported instances of substance use, toxin exposure, and health complications during pregnancy. Anxiety and depression symptoms during pregnancy were assessed via a shortened version of the BAI and the Beck Depression Inventory (Beck and Steer, 1993). A pregnancy screener asked about medical aspects of pregnancy. Overall risk status for the fetus was calculated as a weighted sum of totals from individual measures (Marceau et al., 2013).

#### Adoption Openness

When infants were 4–9 months old, birth and adoptive parents reported the degree of openness in their adoption process. The adoption openness measure included three subscales that were completed by each birth and adoptive parent: perceived openness, contact, and knowledge about birth/adoptive parents.

To score *perceived openness*, birth mothers rated, on a 7-point scale, the degree to which they perceived their adoption process to be open (*very closed* = no information about adoptive, *very open* = semi-regular communication with adoptive parents). Adoptive mothers and fathers provided a global rating of the degree to which they would describe the openness of their adoption (*closed* = no direct contact with a birth parent, *pretty open* = somewhat regular contact with a birth parent). Initial responses to this item for adoptive parents were followed with probes for more detailed descriptions of adoptive parents’ experiences (Ge et al., 2008).

Ratings were standardized and aggregated across raters and measures in order to control for knowledge about birth parents resulting from contact between parties that may influence parent ratings (Ge et al., 2008). This composite was used to control for possible similarities between birth and adoptive parents resulting from contact between families.

### Plan for Analysis

First, in order to minimize the number of terms in the analysis, covariates were regressed out of infant negativity composites. Second, covariance matrices of observed variables were estimated using THEIL (Molenaar, 1995), a FORTRAN program for robust covariance matrix estimation that uses a full information maximum likelihood procedure to adjust for missing data. Third, covariance matrices from THEIL were input into LISREL 8.8 (SSI; Lincolnwood, IL, USA) and SEMs were examined sequentially according to the suggestions of Jöreskog (1971). Initially, measurement model parameters were examined separately for adoptive mothers and adoptive fathers. When fit was adequate, the fit of a two-group model testing adoptive mothers and fathers simultaneously was examined. Given an adequate fit of the two-group model, invariance of factor loadings between adoptive mothers and fathers was examined.

Having established measurement model fit and invariance, a SEM was estimated to test study hypotheses. Fully saturated correlation and regression models were examined; paths that did not achieve at least marginal significance (*p* < 0.10) were removed to create final models. Based on potential limitations of the chi-square (χ^2^) test of exact fit, multiple fit indices were examined. Model fit was considered adequate if RHO (Tucker and Lewis, 1973), the comparative fit index (CFI; Bentler, 1990) and the root-mean square error of approximation (RMSEA; Browne and Cudeck, 1993) met previously established guidelines (Hu and Bentler, 1999).

## Results

### Descriptive Statistics

The number of participants with complete data is shown in **Table 1** along with descriptive statistics. In general, adoptive mothers reported more anxiety symptoms than did adoptive fathers. A repeated-measures ANOVA indicated significant declines in anxiety symptoms over time for both adoptive mothers (*F*_lin_ = 4.54, *p* < 0.05) and adoptive fathers (*F*_lin_ = 28.58, *p* < 0.01). A separate repeated-measures ANOVA revealed no differences in infant negative affect from 9 to 27 months (*F*_1,330_ = 0.02, *p >* 0.10).

**Table 1:** Descriptive Statistics.

	*n*	Mean	*SD*	Min	Max
**Birth parent negative affect**					
Negative affect (18 months)	290	4.08	0.74	1.62	6.19
**Adoptive mother anxiety symptoms**					
Total anxiety symptoms (9 months)	346	3.81	3.58	0.00	17.00
Total anxiety symptoms (18 months)	335	3.43	3.68	0.00	28.00
Total anxiety symptoms (27 months)	325	3.45	4.09	0.00	31.00
**Adoptive father anxiety symptoms**					
Total anxiety symptoms (9 months)	335	3.09	3.08	0.00	19.00
Total anxiety symptoms (18 months)	322	2.36	3.34	0.00	23.00
Total anxiety symptoms (27 months)	310	2.25	2.95	0.00	16.00
**Infant negative affect**					
Negative affect (9 months)	347	-0.01	0.70	-1.32	2.76
Negative affect (18 months)	342	0.00	0.70	-1.65	1.68
Negative affect (27 months)	327	-0.01	0.70	-1.95	1.89

As shown in **Table 2**, anxiety symptoms in adoptive mothers and in adoptive fathers were highly correlated across assessments. Correlations for anxiety symptoms among birth parents, adoptive mothers, and adoptive fathers were small in magnitude. Child negative affect was significantly correlated across assessments and was associated with adoptive fathers’ anxiety symptoms at 9 and 18 months and with adoptive mother anxiety symptoms at 9 months.

**Table 2:** Bivariate correlations.

	1	2	3	4	5	6	7	8	9
**Birth parent negative affect**									
(1) 18 months									
**Adoptive mother anxiety symptoms**									
(2) 9 months	0.02								
(3) 18 months	-0.02	**0.57^∗∗^**							
(4) 27 months	-0.01	**0.53^∗∗^**	**0.56^∗∗^**						
**Adoptive father anxiety symptoms**									
(5) 9 months	0.05	0.06	0.01	0.03					
(6) 18 months	-0.01	0.03	0.10^†^	0.06	**0.56^∗∗^**				
(7) 27 months	0.03	0.10	-0.02	0.07	**0.58^∗∗^**	**0.62^∗∗^**			
**Infant negative affect**									
(8) 9 months	0.03	0.03	0.04	0.11^†^	0.07	0.04	**0.14^∗^**		
(9) 18 months	-0.05	**0.11^∗^**	0.02	0.04	**0.13^∗^**	0.**19^∗∗^**	**0.18^∗∗^**	**0.37^∗∗^**	
(10) 27 months	-0.06	0.10^†^	0.03	0.00	0.10^†^	0.07	0.08	**0.28^∗∗^**	**0.50^∗∗^**

*^∗∗^p < 0.01, ^∗^p < 0.05, ^†^p < 0.10.*

### The Measurement Model

**FIGURE 1:**
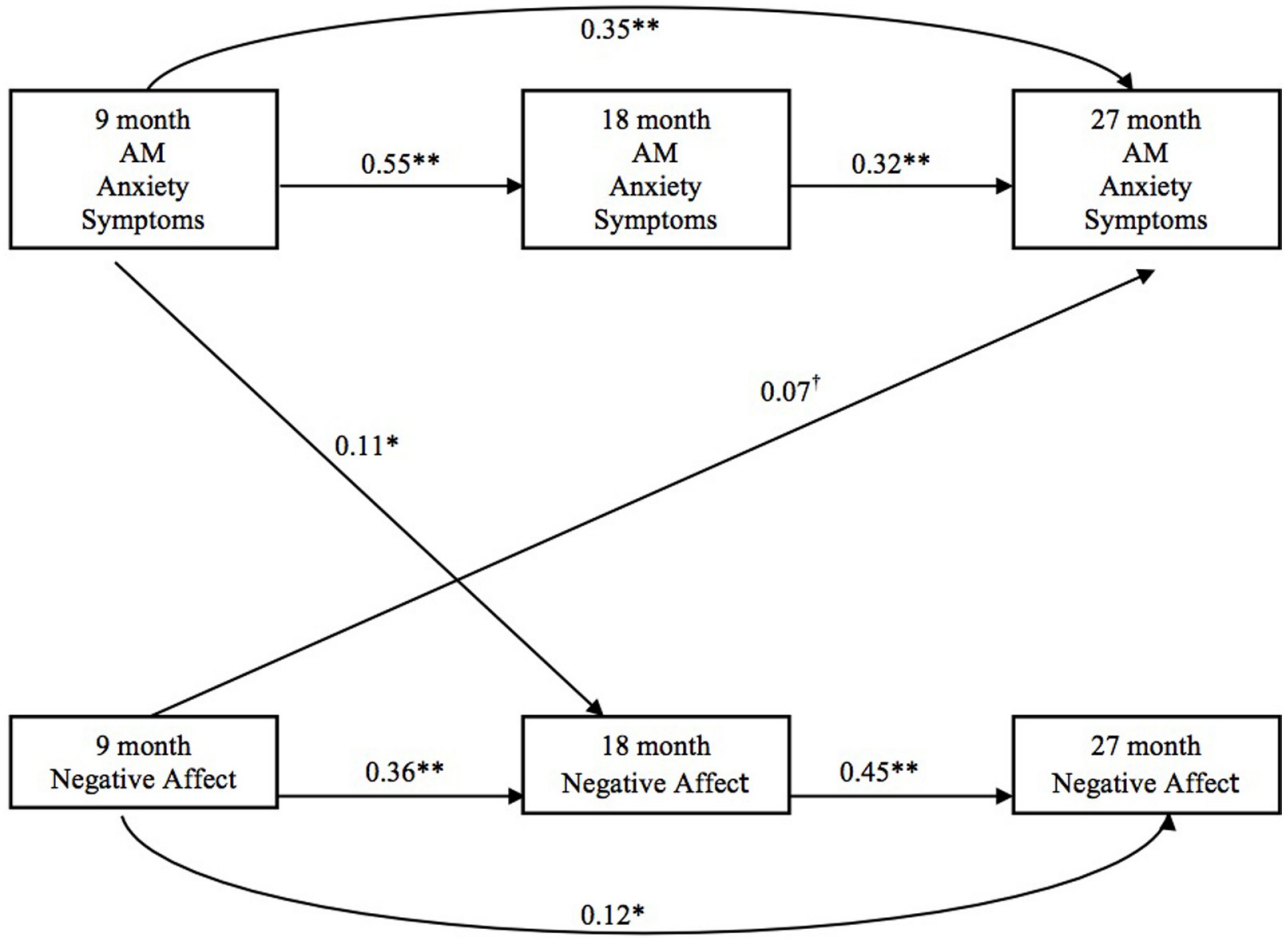
**Infant negative affect and adoptive mother anxiety**.

**FIGURE 2:**
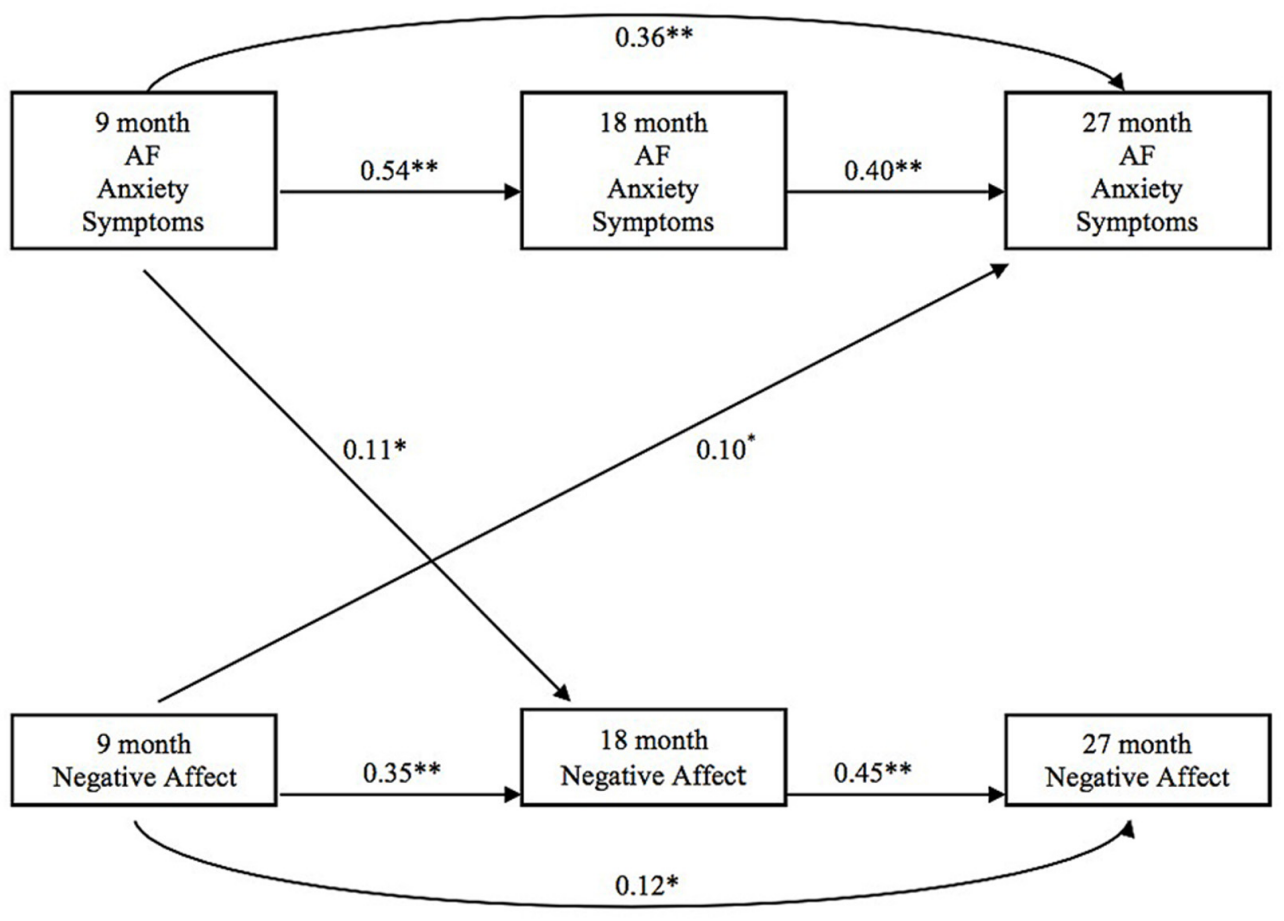
**Infant negative affect and adoptive father anxiety**.

Given that each latent variable was based on a single manifest composite measure, factor loadings were fixed at 1.00 and residual variances for latent factors were not estimated. In addition, in order to underscore the dependence of each latent construct on a single manifest variable, we have illustrated all variables as manifest variables in **Figures 1** and **2**.

First, measurement models were tested separately for adoptive mothers and fathers. The χ^2^ (**Table 3**) was not statistically significant in the final measurement model for adoptive mothers but was marginally significant in the final measurement model for adoptive fathers (*p* < 0.05). However, in large samples, even trivial deviations from a perfect model may be statistically significant (Bentler and Bonett, 1980). Thus, three additional indices were used to inform judgments about model fit (CFI, RMSEA, and RHO). Based on the overall pattern of the fit indices (**Table 3**), model fit was judged as acceptable for both mothers and fathers.

**Table 3:** Model fit indices.

Model	*df*	χ*^2^*	*p*	CFI	RMSEA	ρ
AM measurement model	15	15.60	0.41	0.99	0.01	0.99
AM independence model	21	464.82				
AF measurement model	15	24.58	0.06	0.98	0.04	0.97
AF independence model	21	547.39				
Two-group measurement model	30	41.03	0.98	0.99	0.03	0.98
Two-group independence model	42	1031.09				

*AF, adoptive fathers; AM, adoptive mothers; CFI, Comparative fit index; RMSEA, Root mean square error of approximation.*

We then tested a two-group model to determine invariance of factor structure across groups. Note that only adoptive mother anxiety and adoptive father anxiety differed across the two models. Based on the overall pattern of the three indices of practical fit, the fit of this model was judged to be good.

### The Structural Model

In the final structural model, birth parent negative affect was not significantly correlated with infant negative affect or adoptive mothers’ anxiety across assessments (**Figure 1**). Higher levels of adoptive mother anxiety at 9 months predicted more infant negative affect at 18 months. More infant negative affect at 9 months predicted more adoptive mother anxiety at 27 months.

Birth parent negative affect also was not significantly related to adoptive fathers’ anxiety (**Figure 2**). Higher levels of adoptive father anxiety at 9 months predicted more infant negative affect at 18 months. More infant negative affect at 9 months predicted more adoptive father anxiety at 27 months.

Based on similar patterns of findings for adoptive mothers and fathers, an additional model was examined that constrained each of the estimated paths in the final model to be equal across groups. This analysis tested whether the magnitude of associations was equivalent for adoptive mothers and fathers. Overall fit was not decreased in the constrained model (Δ*df* = 8, Δ*X*^2^ = 0.98, *p* > 0.05), suggesting that the association between infant negative affect and parent anxiety symptoms was equivalent for adoptive mothers and fathers. Notably, in the constrained model, the path linking infant negative affect at 9 months to parent anxiety symptoms at 27 months remained statistically significant (*B* = 0.41, *SE* = 0.14, β = 0.08, *p* < 0.01).

## Discussion

Consistent with previous research, maternal and paternal anxiety symptoms predicted more negative affect in infants 9 months later. Using a longitudinal, multi-trait, multi-method approach, findings are consistent with previous work indicating that more parental anxiety symptoms place children at greater risk for developing their own problems with anxiety (Beidel and Turner, 1997). We provided a novel extension of this work by showing that infants’ negative affect at age 9 months was positively associated with anxiety symptoms in adoptive parents 18 months later. Our results underscore the notion that the added challenge of caring for a more negative infant can be anxiety provoking for parents. This provides some of the first evidence for infant-based effects on parent anxiety symptoms during early childhood. Perhaps most importantly, our work demonstrates that infants’ negative affect and parent anxiety symptoms may jointly contribute to long-term outcomes in both parents and children. We highlight four key insights below.

First, infant negative affect was unrelated to concurrent adoptive parent anxiety symptoms at 9 months, suggesting that associations between child characteristics and parent symptoms may unfold over time. At least one prior study has shown that child age moderates associations between infant characteristics and parent behaviors (Crockenberg, 1986). Similarly, longitudinal relations between parent behaviors and child oppositional behaviors are stronger than concurrent associations (Shaw et al., 1998). In both cases, it is possible that parents initially invest much time and energy into calming and soothing highly negative children, but are unable to sustain such efforts over time (Field et al., 1987). Thus infant-based effects on parent anxiety symptoms may persist or increase over time, making children’s negative affect an increasingly salient factor for anxiety-related outcomes in both children and parents.

Second, to the extent that environments characterized by parental anxiety are detrimental for children’s development (Warren et al., 2003), infant-driven anxiety symptoms in caregivers likely exacerbate the risk for anxiety problems that is already present for infants high in negative affect. Moreover, based on evidence that children with multiple anxious parents are at compounded risk for anxiety problems (Dierker et al., 1999), our finding that greater infant negative affect predicts more anxiety symptoms in both mothers and fathers suggests an even greater likelihood that highly negative infants may be on a trajectory toward their own problems with anxiety. Such an interpretation should be made cautiously given relatively low levels of anxiety symptoms in this typically developing sample. However, the possibility that highly negative infants may be contributing to their own high-risk status by eliciting parent symptoms, which then reinforce the child’s risk trajectory, warrants further exploration.

Finding significant reciprocal influences between negative affect in children and anxiety symptoms in parents underscores the long-held theoretical notion that influences on early development are bidirectional. Specifically, that the direction of effects between a developing person and his/her environment will vary over time is a central tenet of the biocological model proposed by Bronfenbrenner more than 30 years ago (Bronfenbrenner, 1979). However, despite integration into theoretical models of development, empirical work has primarily focused on the unidirectional effect that parents have on child outcomes (Pardini, 2008). Our findings now corroborate work from other domains suggesting that developmental models that account only for parent influences on children’s development are inadequate for understanding the true nature of development. Indeed, consistent with a broader literature on the importance of evocative effects (Scarr and McCartney, 1983), our work suggest that children are active participants in constructing their early environments, even when environmental characteristics may prove detrimental to child outcomes. The implication of such findings is that intervention and prevention efforts that focus solely on either child behaviors or parent symptoms are not fully addressing the scope of early risk. This will be a critical factor that will need to be addressed as research on early anxiety risk progresses.

Third, although more negative affect in infants was related to higher levels of anxiety symptoms in parents over time, mean levels of anxiety symptoms in adoptive parents tended to decline overall. This raises the possibility that the relation between infant negative affect and parent anxiety is self-limiting, or that negative affect in children is linked to limited declines in parent symptoms rather than increases in parent symptoms. That is, normative reductions in anxiety symptoms in parents that typically occur as children develop in early childhood may be stymied or reduced for parents with highly negative children. It should be kept in mind, however, that small-scale changes in parent anxiety symptoms may be obscured by the use of standardized measures in this report.

Our results cannot speak to long-term mental health outcomes for birth parents. Previous research does, in fact, suggest that relinquishing an infant for adoption may impact long-term adjustment in adoptive mothers (Brodzinsky, 1990). The extent to which the development of anxiety symptoms in birth mothers may be mitigated or enhanced by environmental factors, such as adoption openness, continues to be debated (Miall and March, 2005). Therefore, future research that extends the current analyses to examine genetic and environmental factors that contribute to longitudinal adjustment in birth parents would greatly contribute to this area of research.

Finally, birth parent negative affect was unrelated to infant negative affect, suggesting that genetic influences as measured in the current study are not driving the effects observed in this work. Previous research with twins has reported heritable influences on both negative affect (Baker et al., 1992) and anxiety symptoms (Hettema et al., 2001). It is possible that our general measures of negative affect in birth parents may be less sensitive than specific measures. However, our results are unchanged when our measure of birth parent negative affect is replaced with measures of clinical anxiety symptoms. Similarly, results are unchanged when only birth mother data are considered. Thus, it is more likely that methodological differences or developmental changes in gene expression account for the lack of genetic effects in the current study. Relative to twin designs, adoption studies are less sensitive in detecting genetic effects, in part because only additive genetic influences between adult birth parents and, in this case, young children are examined. Previous work has suggested that an absence of genetic effects that may result from fluctuations in the genetic influence most relevant to anxiety risk as children develop (Eley et al., 2015). Alternatively, it may also be the case that genetic effects on negative affect in offspring are muted in the context of family stability and/or ample resources in the early environment.

The current study is not without limitations. The variability in anxiety symptoms appears greater for adoptive parents over time, which increases the likelihood that children’s negative affect will significantly predict adoptive parent symptoms at later, rather than earlier assessments. Little is known about the normative trajectories of anxiety symptoms in adoptive families. Because of this, it is difficult to understand whether increased variability at later assessments is characteristic of the development of anxiety symptoms in adoptive parents or is unique to these data.

Additionally, adoptive parent anxiety symptoms were not measured via clinical interview. Although a range of anxiety symptoms was present, only a small percentage of adoptive parents met BAI criteria for moderate (∼2%) and severe (∼2%) levels of anxiety. Subthreshold anxiety levels, as measured by the BAI, adequately characterize levels of impairment in adults and predict subsequent diagnoses (Karsten et al., 2011). Additionally, based on the young age of our child participants, when diagnoses are rare, the degree to which these associations predict subsequent diagnoses in children is not yet known. Thus, this study must be considered an investigation of early contributors to childhood risk rather than an assessment of anxious children.

To our knowledge, this work provides the first evidence for bidirectional effects between infant negative affect and parents’ anxiety symptoms during infancy. Although additional work is needed to replicate findings and investigate developmental mechanisms, these results offer insight about the roles of infant negative affect and parent anxiety symptoms that may enhance our ability to identify, intervene, and treat children at risk for elevated symptomatology.

## Conflict of Interest Statement

The authors declare that the research was conducted in the absence of any commercial or financial relationships that could be construed as a potential conflict of interest.
